# Gold Nanoparticles Affect Pericyte Biology and Capillary Tube Formation

**DOI:** 10.3390/pharmaceutics13050738

**Published:** 2021-05-17

**Authors:** Sasikarn Looprasertkul, Amornpun Sereemaspun, Nakarin Kitkumthorn, Kanidta Sooklert, Tewarit Sarachana, Depicha Jindatip

**Affiliations:** 1 Department of Anatomy, Faculty of Medicine, Chulalongkorn University, 1873 Rama 4 Rd., Wangmai, Pathumwan, Bangkok 10330, Thailand; sasikarn.loo@alumni.chula.ac.th (S.L.); amornpun.s@chula.ac.th (A.S.); kanidta.s@chula.ac.th (K.S.); 2 Nanomedicine Research Unit, Department of Anatomy, Faculty of Medicine, Chulalongkorn University, Bangkok 10330, Thailand; 3 Department of Oral Biology, Faculty of Dentistry, Mahidol University, Payathai Rd., Ratchathewi, Bangkok 10400, Thailand; nakarin.kit@mahidol.ac.th; 4 Age-Related Inflammation and Degeneration Research Unit, Department of Clinical Chemistry, Faculty of Allied Health Sciences, Chulalongkorn University, 154 Rama 1 Rd., Wangmai, Pathumwan, Bangkok 10330, Thailand; tewarit.sa@chula.ac.th; 5 SYstems Neuroscience of Autism and PSychiatric Disorders (SYNAPS) Research Unit, Department of Clinical Chemistry, Faculty of Allied Health Sciences, Chulalongkorn University, Bangkok 10330, Thailand; 6 Division of Histology and Cell Biology, Department of Anatomy, School of Medicine, Jichi Medical University, 3311-1 Yakushiji, Shimotsuke, Tochigi 329-0498, Japan

**Keywords:** angiogenesis, capillary tube formation, endothelial cells, gold nanoparticles, pericytes

## Abstract

Gold nanoparticles (AuNPs) are used for diagnostic and therapeutic purposes, especially antiangiogenesis, which are accomplished via inhibition of endothelial cell proliferation, migration, and tube formation. However, no research has been performed on the effects of AuNPs in pericytes, which play vital roles in endothelial cell functions and capillary tube formation during physiological and pathological processes. Therefore, the effects of AuNPs on the morphology and functions of pericytes need to be elucidated. This study treated human placental pericytes in monoculture with 20 nm AuNPs at a concentration of 30 ppm. Ki-67 and platelet-derived growth factor receptor-β (PDGFR-β) mRNA expression was measured using real-time reverse transcription-quantitative polymerase chain reaction (RT-qPCR). Cell migration was assessed by Transwell migration assay. The fine structures of pericytes were observed by transmission electron microscopy. In addition, 30 ppm AuNP-treated pericytes and intact human umbilical vein endothelial cells were cocultured on Matrigel to form three-dimensional (3D) capillary tubes. The results demonstrated that AuNPs significantly inhibited proliferation, reduced PDGFR-β mRNA expression, and decreased migration in pericytes. Ultrastructural analysis of pericytes revealed AuNPs in late endosomes, autolysosomes, and mitochondria. Remarkably, many mitochondria were swollen or damaged. Additionally, capillary tube formation was reduced. We found that numerous pericytes on 3D capillary tubes were round and did not extend their processes along the tubes, which resulted in more incomplete tube formation in the treatment group compared with the control group. In summary, AuNPs can affect pericyte proliferation, PDGFR-β mRNA expression, migration, morphology, and capillary tube formation. The findings highlight the possible application of AuNPs in pericyte-targeted therapy for antiangiogenesis.

## 1. Introduction

Recently, nanoparticles have been developed for innovative therapeutic strategies involving nanotechnology. Nanoparticles are small particles with diameters of diverse sizes from 1 to 100 nm. Notably, gold nanoparticles (AuNPs), which are some of the most common metal nanoparticles, have been widely used in nanomedicine. The remarkable properties of AuNPs are their low cytotoxicity, good biocompatibility, and ease of conjugation to a variety of biomolecules [1,2]. At present, AuNPs are used for cell imaging, targeted drug delivery, thermal therapy, antiangiogenesis, and anticancer applications [3,4,5]. Recent studies have reported that AuNPs ranging from 15 to 30 nm in size can aid in antiangiogenesis by inhibiting the activity of human umbilical vein endothelial cells (HUVECs) [1,6,7]. For example, 15 nm AuNPs inhibit migration and tube formation [6]. In addition, 20 nm AuNPs inhibit proliferation and tube formation and reduce phosphorylation-mediated activation of the extracellular signal-regulated kinase 1/2 (ERK 1/2), protein kinase B (Akt), and focal adhesion kinase (FAK) signaling pathways in vascular endothelial growth factor (VEGF)165-induced HUVECs [1,6]. Similarly, 26 nm AuNPs reduce cell viability, cell proliferation, cell migration, and tube formation and increase the expression of autophagy genes, including autophagy related 5 (ATG5), beclin1 (an autophagosome formation marker), p62 (a degradation marker), and microtubule-associated protein 1 light chain 3 (LC3)-II (an autophagy activation marker), in HUVECs [7]. Apart from inhibiting HUVEC activity, AuNPs can suppress angiogenesis in choroidal neovascularization-induced age-related macular degeneration in vivo [1]. Moreover, AuNPs have been found to inhibit oxygen-induced retinopathy by reducing both neovascular and avascular areas in an in vivo retinal neovascularization study [7].

The formation, maintenance, and remodeling of capillaries are accomplished by cooperative activity between endothelial cells and pericytes. Pericytes are perivascular cells located at the abluminal surfaces of small, thin vessels, i.e., capillary and postcapillary venules, and share a basement membrane with endothelial cells [8]. Pericytes are responsible for regulating vascular maturation, stabilizing blood vessels, and controlling blood flow, capillary diameter, and hydrostatic balance [9,10,11]. In addition, pericytes can promote endothelial cell survival and migration through paracrine signaling, such as platelet-derived growth factor-BB (PDGF-BB)-platelet-derived growth factor receptor-β (PDGFR-β) signaling [12]. This signaling is essential to endothelial cells and is also crucial for pericyte survival, proliferation, migration, recruitment, attachment, and differentiation [13]. Previous reports have indicated that a lack of PDGF or PDGFR-β expression prevents pericyte recruitment to vessels [14,15]. Recently, pericytes have been discovered to be targets in antiangiogenesis mediated by blockade of PDGFR-β [16].

As mentioned above, there have been studies on the use of AuNPs for antiangiogenesis via endothelial cell targeting. However, the capillary networks formed during angiogenesis require two cell types: endothelial cells and pericytes. Therefore, we aimed to determine whether AuNPs might also affect pericyte properties. In this study, the effects of AuNPs on pericyte morphology, proliferation, migration, and PDGFR-β mRNA expression were elucidated. Additionally, AuNP-treated pericytes were cocultured with intact endothelial cells to discover the characteristics of three-dimensional (3D) capillary tube formation.

## 2. Materials and Methods

### 2.1. AuNP Characterization

Stable suspensions of 20 nm-diameter AuNPs (Cat. no. 741965, Sigma-Aldrich, St. Louis, MO, USA) in citrate buffer were used in this study. The shapes and sizes of the AuNPs were confirmed by transmission electron microscopy (TEM) (JEM-1400PLUS; JEOL, Tokyo, Japan). In addition, the ultraviolet–visible (UV-Vis) absorption of the AuNPs was evaluated using a microplate spectrophotometer (Multiskan GO; Thermo Scientific, Vantaa, Finland).

### 2.2. Cell Culture

Human placental pericytes (hPC-PLs; Cat. no. C-12980, PromoCell, Heidelberg, Germany) were cultured in pericyte growth medium (Cat. no. C-28040, PromoCell, Heidelberg, Germany) supplemented with 10% SupplementMix (Cat. no. C-39840, PromoCell, Heidelberg, Germany) and 1% antibiotic-antimycotic solution (Cat. no. 15240062, Gibco, New York, NY, USA). HUVECs (Cat. no. C2519A, Lonza, Basel, Switzerland) were cultured in endothelial cell growth medium supplemented with EGM-2MV SingleQuots (Cat. no. CC-4147, Lonza, Basel, Switzerland) and 1% antibiotic-antimycotic solution (Cat. no. 15240062, Gibco, New York, NY, USA). Both cell types were maintained at 37 °C in a humidified 5% CO_2_ atmosphere.

### 2.3. Cell Viability Assay

Pericytes (1 × 10^4^ cells/well) were seeded into 96-well plates and incubated overnight. To determine the optimal dose for the induction of cytotoxicity, pericytes were treated with 20 nm AuNPs at various concentrations, i.e., 10, 20, 30, 40, and 50 ppm, for 24 h. PrestoBlue (Cat. no. A13262, Invitrogen, New York, NY, USA) was used according to the manufacturer’s protocol. After adding the reagent and incubating for 4 h, pericyte viability was determined by measuring the UV absorbance using a microplate reader at 570/600 nm.

### 2.4. Real-Time Reverse Transcription-Quantitative Polymerase Chain Reaction (Real-Time RT-qPCR)

According to the cell viability assay, the 30 ppm concentration was selected for the following experiments because it was the highest dose at which pericytes remained viable (over 90%). Pericytes were seeded into 24-well plates at a density of 1 × 10^5^ cells/well. The cells were treated with 30 ppm AuNPs for 24 h. Total RNA was isolated from the pericytes with TRIzol reagent (Cat. no. 15596026, Invitrogen, Carlsbad, CA, USA) and reverse-transcribed into cDNA with a RevertAid First-Strand cDNA Synthesis Kit (Cat. no. K-1622, Thermo Fisher Scientific Inc., Rockford, IL, USA). Next, real-time RT-qPCR was carried out using AccuPower 2X Greenstar qPCR Master Mix (Cat. no. K-6254, Bioneer, Daejeon, Korea) in a volume of 12.5 μL with a CFX 96 thermocycler (Bio-Rad Laboratories, Hercules, CA, USA). The mRNA expression of PDGFR-β, a known pericyte marker, was examined with forward (5′-ACTGCCCAGACCTAGCAGTG-3′) and reverse (5′-CAGGGAAGTAAGGTGCCAAC-3′) primers. Moreover, Ki-67 mRNA expression was analyzed to detect cell proliferation using forward (5′-GATCGTCCCAGTGGAGTT-3′) and reverse (5′-CCCCTTCCAAACAAGCAGGT-3′) primers. GAPDH was used as a housekeeping gene.

### 2.5. Transwell Migration Assay

A cell migration assay was performed using 8 µm pore size Transwell inserts (Corning Inc., New York, NY, USA). After pretreatment with 30 ppm AuNPs for 24 h, pericytes (200 µL, 2.5 × 10^4^ cells-treated/well) were plated on the upper chamber and cultured in serum-free culture medium. In the lower chamber, culture medium containing PDGF-BB (Cat. no. 220-BB, R&D Systems, Minneapolis, MN, USA) and 10% FBS (Cat. no. 1600044, Gibco, Carlsbad, CA, USA) was used as a chemoattractant. The cells were incubated for 48 h. Migrating cells that moved to the lower surface of the membrane were fixed with 4% paraformaldehyde (Cat. no. 818715.1000, Merck Millipore, Darmstadt, Germany) for 10 min and then stained with 1% crystal (Cat. no. 03050–25, Loba Chemie, Mumbai, India) violet in 2% ethanol (Cat. no. 1.000983.2500, Merck Millipore, Darmstadt, Germany) for 20 min. Nonmigrating cells in the upper chamber were removed with a cotton swab and then washed with distilled water. The migrating cells were imaged by inverted light microscopy (CKX53, Olympus, Tokyo, Japan) and counted with ImageJ software version 1.52a (Wayne Rasband, National Institutes of Health, Bethesda, MD, USA).

### 2.6. Transmission Electron Microscopy

AuNP-treated pericytes were fixed with 2.5% glutaraldehyde (Cat. no. 16220, EMS, Hatfield, PA, USA) at 4 °C for 2 h and postfixed with 1% osmium tetroxide (Cat. no. 19110, EMS, Hatfield, PA, USA) at 4 °C for 1.5 h. The cell pellets were dehydrated with an alcohol series (Cat. no. 1.000983.2500, Merck Millipore, Darmstadt, Germany) and cleared by gradual addition of propylene oxide (Cat. no. 8.07027.1001, Merck Millipore, Darmstadt, Germany). Then, the pellets were embedded in epoxy resin and cut into slices of 70 nm thickness with an ultramicrotome (EM UC7, Leica, Vienna, Austria). The ultrathin sections were stained with uranyl acetate and lead citrate and observed by transmission electron microscopy (JEM-1400PLUS; JEOL, Tokyo, Japan).

### 2.7. Capillary Tube Formation Assay

To coculture pericytes and endothelial cells for capillary tube formation, 100 µL of Matrigel (Cat. no. 356231, Corning, Bedford, MA, USA) was added to an 8-chamber slide, and the slide was incubated at 37 °C for 30 min for Matrigel solidification. AuNP-treated pericytes and intact endothelial cells were seeded and cocultured at a ratio of 1:20 on the 8-chamber slide. All tubes in the control and 30 ppm AuNP-treated groups were quantified by manual cell counting with the aid of ImageJ software version 1.52a under an inverted light microscope (CKX53, Olympus, Tokyo, Japan).

### 2.8. Immunofluorescence Histochemistry of Pericytes during Tube Formation

Capillary tubes from pericyte/endothelial cell coculture were fixed with 4% paraformaldehyde (Cat. no. 818715.1000, Merck Millipore, Darmstadt, Germany) at 4 °C for 20 min. Nonspecific binding on the tubes was blocked with 2% normal goat serum (Cat. no. S-1000, Vector Laboratories, Burlingame, CA, USA) in PBS at 30 °C for 20 min. The tubes were then incubated with a monoclonal rabbit IgG anti-PDGFR-β antibody (diluted 1:100, 28E1, Cat. no. 3169S, Cell Signaling, Danvers, MA, USA) overnight at 4 °C followed by an Alexa Fluor 488 goat anti-rabbit IgG conjugated secondary antibody (diluted 1:200, Cat. no. A-11008, Invitrogen, Paisley, UK) at 30 °C for 30 min. The nuclei were stained with Prolong Diamond Antifade Mountant with DAPI (Cat. no. P36962, Invitrogen, Eugene, OR, USA). The morphology and location of pericytes were observed with a confocal laser scanning microscope (LSM900, ZEISS, Oberkochen, Germany) and analyzed with Imaris software version 9.3.1 (Bitplane, Belfast, UK).

### 2.9. Statistical Analysis

The data are expressed as the mean ± SEM from three independent experiments. Student’s t-test was conducted to compare two groups, whereas one-way ANOVA and Tukey’s post hoc test were used to compare multiple groups. Statistical analyses were carried out using GraphPad Prism 5.01 (GraphPad Software, Inc., San Diego, CA, USA) and SPSS 23.0 (SPSS, Inc., Armonk, NY, USA). Differences with a value of *p* < 0.05 were considered statistically significant.

## 3. Results

### 3.1. Characterization of the AuNP

**Figure 1:**
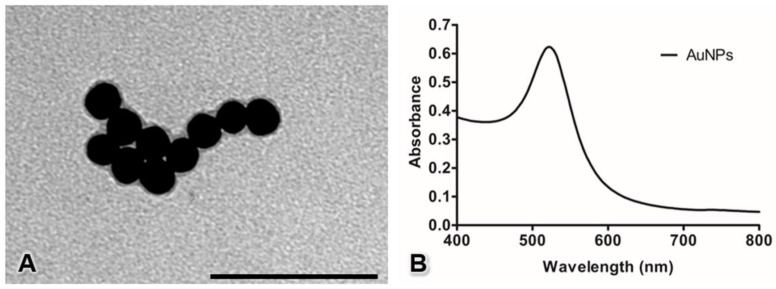
Characterization of AuNPs by transmission electron microscopy (**A**) and UV-Vis spectroscopy (**B**). Bar: 100 nm.

The AuNPs observed under TEM revealed a spherical morphology with an average diameter of 20.04 ± 0.13 nm (Figure 1A). The UV-Vis spectrum of 20 nm-diameter AuNPs showed a single wavelength peak at approximately 522 nm (Figure 1B).

### 3.2. Pericyte Viability

**Figure 2:**
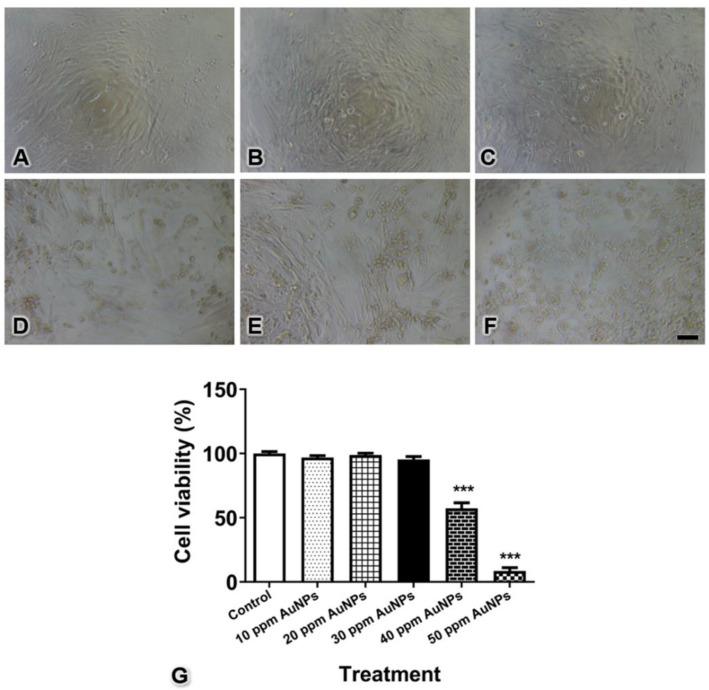
Cell viability of control pericytes (**A**) and pericytes treated with AuNPs at 10 (**B**), 20 (**C**), 30 (**D**), 40 (**E**), and 50 ppm (**F**) concentrations. The graph shows the quantitative data for pericyte viability (**G**). *** *p* < 0.001. Bar: 100 μm.

To select the optimal concentration of 20 nm AuNPs in the present study, the effects of AuNPs on pericyte viability were assessed. The morphological changes upon the treatment of the cells with 20 nm AuNPs are shown in Figure 2A–F. The quantitative results showed that no difference was detectable between the control pericytes and the pericytes treated with 10, 20, and 30 ppm AuNPs, but pericytes treated with AuNPs at concentrations higher than 40 ppm exhibited significantly reduced cell viability (Figure 2G, *** *p* < 0.001). Notably, the sizes and shapes of AuNP-treated pericytes started to change when an AuNP concentration of 30 ppm was used, as observed under microscopy. Therefore, we selected a concentration of 30 ppm for the next experiments.

### 3.3. Ki-67 and PDGFR-β mRNA Expression

**Figure 3:**
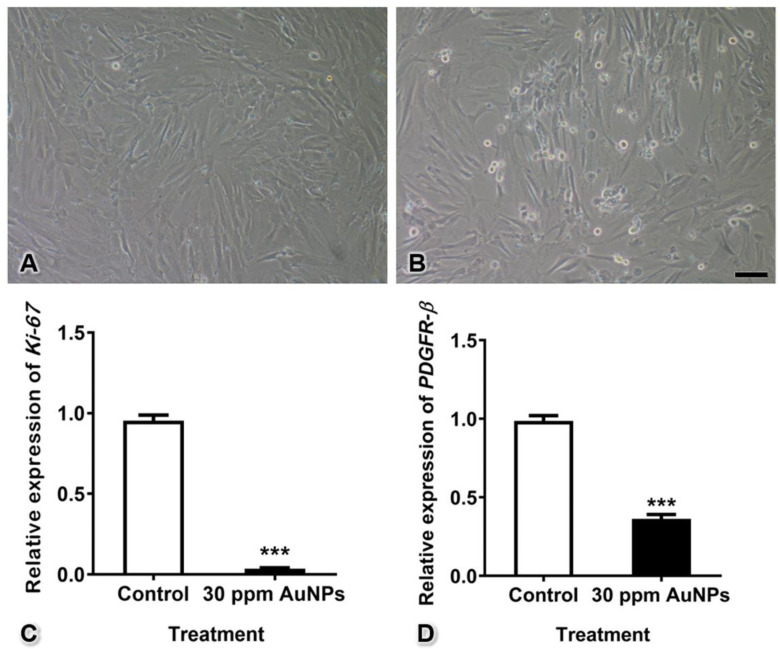
Pericyte proliferation and PDGFR-β mRNA expression after treatment with 30 ppm AuNPs for 24 h. Representative images are shown for the control group (**A**) and the 30 ppm AuNP-treated pericyte group (**B**). The graphs show the Ki-67 (**C**) and PDGFR-β (**D**) mRNA expression levels. *** *p* < 0.001. Bar: 100 nm.

The relative mRNA expression of Ki-67, a marker of cell proliferation, was significantly decreased after treatment with 30 ppm AuNPs (fold change of control = 0.955 ± 0.034; 30 ppm AuNPs = 0.037 ± 0.004). In addition, the relative mRNA expression of PDGFR-β, a marker of pericytes, was also decreased in the treatment group compared with the control group (fold change of control = 0.987 ± 0.033; 30 ppm AuNPs = 0.363 ± 0.028) (Figure 3, *** *p* < 0.001).

### 3.4. Pericyte Migration

**Figure 4:**
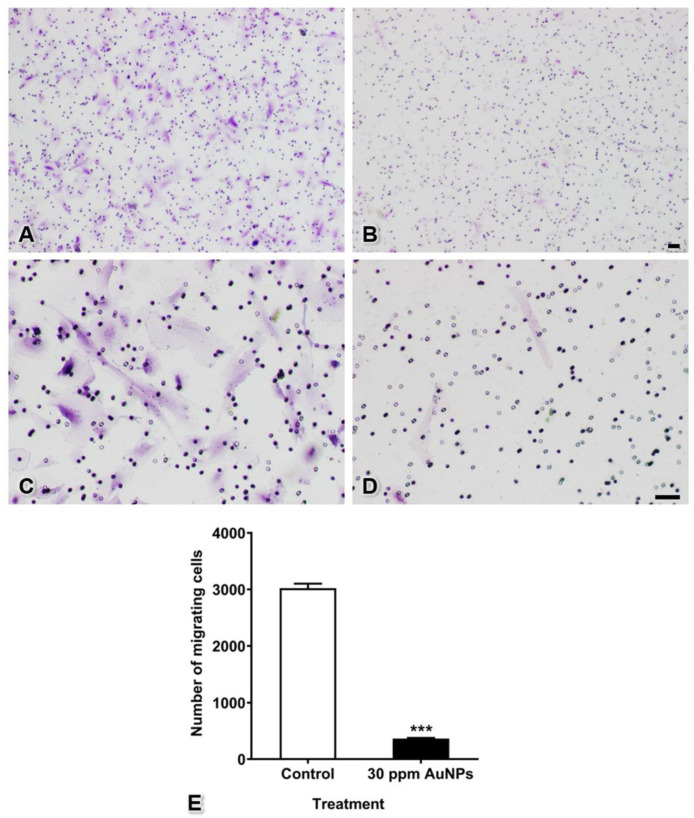
Cell migration of pericytes after treatment with 30 ppm AuNPs. Representative images are shown for the control group (**A**,**C**) magnified view of (**A**) and the 30 ppm AuNP-treated pericyte group (**B**,**D**) magnified view of (**B**). The graph shows the numbers of migrating cells (**E**). *** *p* < 0.001. Bars: 100 μm.

Migrating cells of both the control and 30 ppm AuNP-treated groups passed through the membrane pores and expanded their cytoplasm onto the lower surface of the Transwell membrane. The results showed that AuNPs markedly reduced the number of cells moving through the membrane (365 ± 10 cells) from the number in the control group (3022 ± 80 cells) (Figure 4, *** *p* < 0.001).

### 3.5. Transmission Electron Microscopy

**Figure 5:**
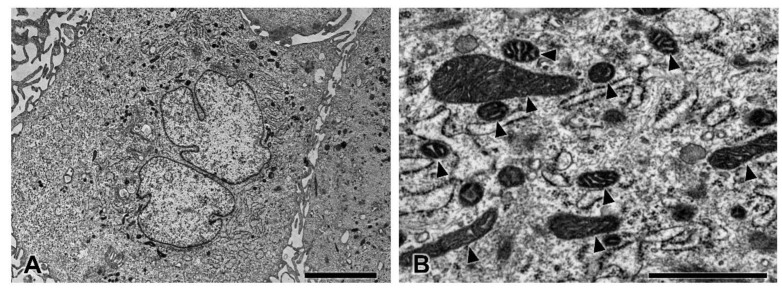
Transmission electron microscopy of pericytes in the control group at low (**A**) and high magnifications (**B**). The pericytes showed normal ultrastructure. Note the healthy mitochondria (arrowhead). Bars: 5000 nm (**A**), 1000 nm (**B**).

**Figure 6:**
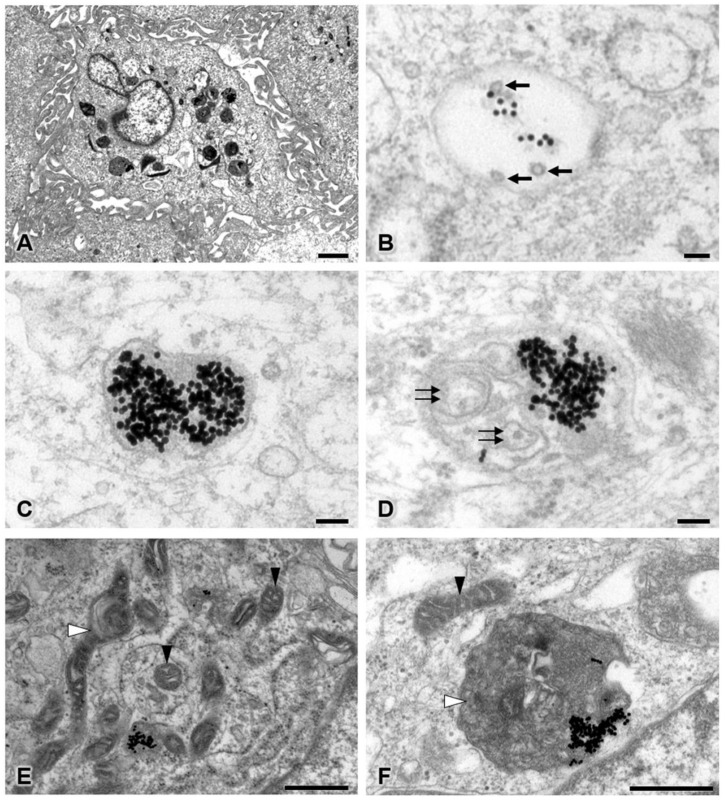
Transmission electron microscopy of the 30 ppm AuNP-treated pericyte group at low (**A**) and high magnifications (**B**–**F**). Representative images are shown for late endosomes containing AuNPs and multivesicular bodies (**B**), autolysosomes containing only AuNPs (**C**), and autolysosomes containing AuNPs and intracellular debris (**D**). Affected mitochondria showed mild to severe swelling (**E**,**F**). Note the AuNPs (electron-dense dots), multivesicular bodies (black arrows), intracellular debris (double black arrows), unaffected mitochondria (black arrowhead), and swollen or damaged mitochondria (white arrowhead). Bars: 1000 nm (**A**), 100 nm (**B**–**D**), 500 nm (**E**,**F**).

To study the intracellular uptake of AuNPs, transmission electron microscopy was used. In the control group, pericytes presented no disruption of the cytoplasmic and nuclear membranes. The mitochondria and endoplasmic reticulum were normal (Figure 5). The plasma membrane, nucleus, and nuclear membrane structures of the 30 ppm AuNP-treated pericytes were similar to those of the control pericytes (Figure 6A). AuNPs were found in late endosomes (Figure 6B), autolysosomes (Figure 6C,D), and mitochondria (Figure 6E,F). Interestingly, mitochondria affected by AuNPs were swollen and damaged (Figure 6E,F). Free AuNPs in the cytosol were not observed. In addition, we treated pericytes with 50 ppm AuNPs, and the results revealed that pericytes were severely damaged and died (Supplemental Figures S1 and S2).

### 3.6. Capillary Tube Formation

**Figure 7:**
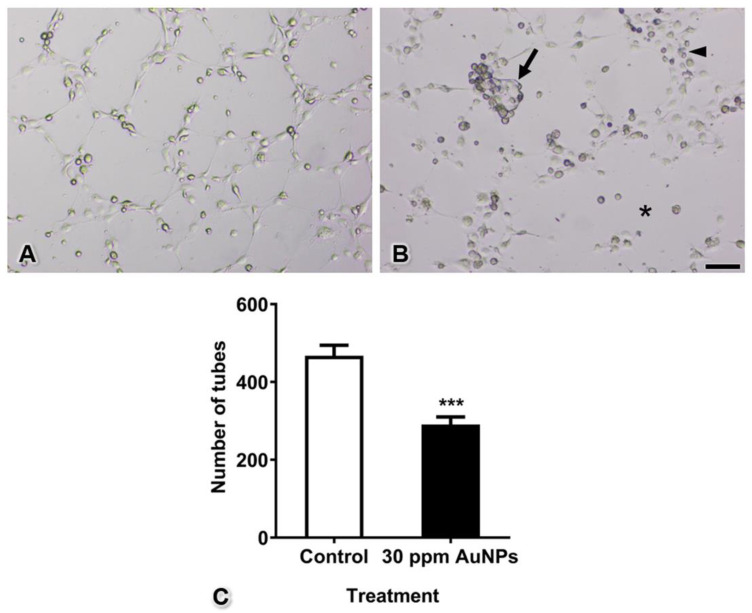
Tube formation of the control group (in which intact endothelial cells were cocultured with intact pericytes) (**A**) and the AuNP-treated group (in which intact endothelial cells and pericytes were pretreated with AuNPs) (**B**). The graph shows the numbers of tubes (**C**). Note the cluster of mixed pericytes and endothelial cells (arrow), the thick wall of tube formation (arrowhead), and the area of incomplete tubes (asterisk). *** *p* < 0.001. Bar: 500 µm.

The tubes that formed in the control group were complete with thin and elongated walls, similar to capillaries in vivo. In the AuNP-treated group, pericytes and endothelial cells did not migrate to form tubes. Completed tubes were rarely found, and some of these tubes presented thicker walls in the AuNP-treated group than in the control group. To quantify tube formation, all completed tubes in the control and AuNP-treated groups were counted after culture for 4 h. The results showed that the number of tubes formed significantly decreased from 467.70 ± 26.76 tubes to 290.80 ± 19.56 tubes (Figure 7, *** *p* < 0.001).

### 3.7. Immunofluorescence Histochemistry of Pericytes during Tube Formation

**Figure 8:**
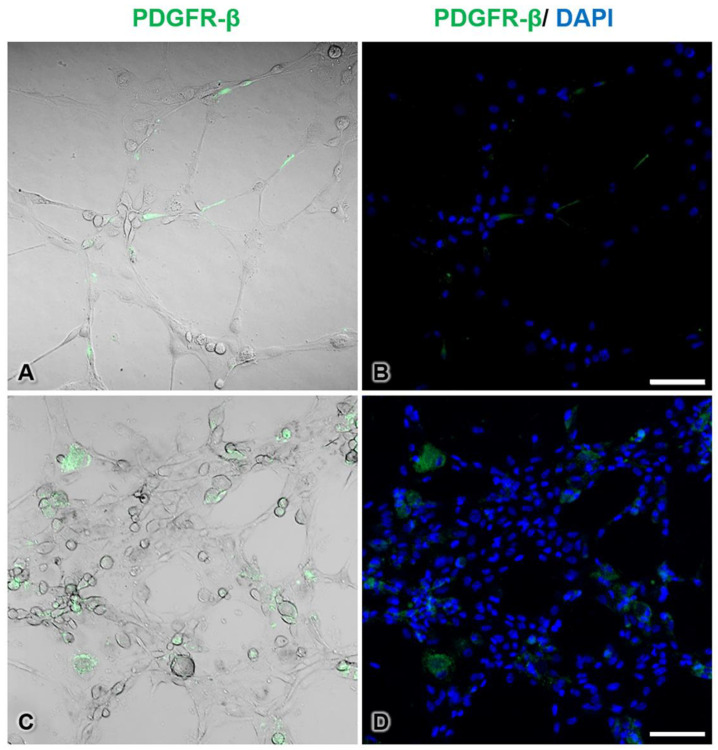
Immunofluorescence staining of pericytes during tube formation in the control group (**A**,**B**) and the 30 ppm AuNP-pretreated pericyte group (**C**,**D**). PDGFR-β-immunopositive cells are pericytes (green pseudocolor), and unstained cells are endothelial cells. In the treated group, pericytes were round and did not extend processes. Note that the endothelial cells and AuNP-treated pericytes formed thicker walls than the control cells. The nuclei are stained with DAPI (blue pseudocolor). Bars: 100 µm.

To observe the morphology and localization of pericytes during tube formation, immunofluorescence histochemical staining for PDGFR-β, which is a marker of pericytes, was conducted. In the control group, pericytes showed intense PDGFR-β-immunopositive staining, had elongated shapes, and were located beside endothelial cells along the tubes (Figure 8A,B). On the other hand, the tube-forming pericytes in the AuNP-treated group appeared round in shape and did not extend processes (Figure 8C,D). In addition, many mixed cell clusters of pericytes and endothelial cells were observed in the treated group.

## 4. Discussion

The objective of this study was to reveal the effects of AuNPs on biological changes in pericytes and capillary tube formation. AuNPs significantly reduced pericyte proliferation, migration and PDGFR-β mRNA expression. Transmission electron microscopy revealed AuNP aggregation within mitochondria, which caused pleomorphic features and mild to severe damage. Moreover, we found that pericytes that were treated with AuNPs formed incomplete capillary tubes even though they were cocultured with intact endothelial cells.

Previous studies have reported that AuNPs larger than 5 nm have a chemically inert character, resulting in low cytotoxicity. AuNPs with a size of 20 nm are more effective than 5 and 10 nm AuNPs in inhibiting HUVEC proliferation [17] and do not induce cytotoxicity in the retinal pigment epithelium [1], retinal microvascular endothelial cells [18], or nonmalignant NIH3T3 fibroblast cells [19]. Moreover, AuNPs of this size are frequently applied in HUVEC tube formation and angiogenesis studies [1,17,20,21]. Previous reports have mentioned that 20 nm AuNPs, especially at relatively low concentrations, can significantly change the biological characteristics of HUVECs; for example, they can reduce cell proliferation, cell migration, and tube formation [1,7]. In our study, 20 nm AuNPs at concentrations of 40 and 50 ppm caused cell death. Thus, 30 ppm, which was the highest concentration that maintained high cell survival, was chosen for further experiments (Figure 2). However, it is worth noting that cellular changes are dependent not only on the size and concentration of nanoparticles but also on the shape, surface charge, dispersion in solvent, exposure duration, molecule coating, and type of cell [4,7,19,22,23,24].

AuNPs can inhibit several endothelial activities and cell survival by changing the conformation of VEGF or VEGF receptor in HUVECs [1,6]. For pericytes, the PDGF-BB-PDGFR-β signaling pathway is essential for proliferation, migration, maturation, differentiation, and survival during capillary tube formation [15,25,26]. PDGF-BB is released from endothelial cells and interacts with PDGFR-β, a receptor on the surfaces of pericytes. This interaction results in autophosphorylation and noncovalent dimerization of specific tyrosine residues in the pericyte cytoplasm. Cell proliferation is a process that occurs through the phosphoinositide-3-kinase (PI3K) pathway, and cell migration is mediated by the Src homology-2 domain-containing protein tyrosine phosphatase-2 (SHP-2)-mediated mitogen-activated protein kinase (MAPK) pathway [13,26]. Therefore, in the present study, we hypothesized that AuNPs could reduce PDGFR-β mRNA expression, leading to decreases in proliferation and migration (Figure 3 and Figure 4) via these pathways.

In our nanostructural study, as shown in Figure 6, AuNPs at 30 ppm did not cause cell death or destroy the plasma membrane, nucleus, or nuclear membrane, but they did cause swelling and damage to mitochondria. AuNPs were mainly engulfed into endosomes that fused with lysosomes containing digestive enzymes (Figure 6A–D) in the typical endocytic pathway [27]. However, a number of AuNPs were observed in mitochondria (Figure 6A,E,F). These nanoparticles were transferred from endosomes through the endosomal escape pathway [28] and might subsequently have been transferred to autolysosomes for degradation. Mitochondrial dysfunction disrupts ATP production, apoptosis regulation, metabolism integration, and calcium homeostasis in cells [29]. The rest of the AuNPs that lysosomes could not digest might have been eliminated by cellular exocytosis [30].

Studies by Roh et al. (2016) and Shen et al. (2018) have shown that AuNPs can inhibit 3D capillary tube formation [1,7]. However, both studies performed capillary tube formation in monocultures of a single cell type: endothelial cells. In contrast, our present study performed tube formation using a 3D coculture method with both endothelial cells and pericytes to imitate the capillary vasculature found in almost all tissues. Moreover, the results shown in Figure 7 and Figure 8 reveal that although endothelial cells were intact, defects in pericytes induced incomplete capillary tube formation. Furthermore, the cell aggregation found in the 3D culture system indicated that a number of cells did not migrate to form tubes, consistent with the results of the migration assay shown in Figure 4. We presume that the tube-guiding property of pericytes [9] is impaired after administration of AuNPs.

## 5. Conclusions

This is the first study on the effects of AuNPs focusing on changes in pericyte characteristics and properties. Moreover, the results from the 3D coculture, which included pericytes in capillary formation, suggest that this cell type could be considered an antiangiogenic target in nanoparticle therapy. Finally, future directions for this research include evaluating the elimination of AuNPs on pericytes in vitro, as well as studying the cytotoxicity and clearance rate of AuNPs in vivo, both of which are essential for developing novel antiangiogenic therapies.

## Supplementary Materials

The following are available online at https://www.mdpi.com/article/10.3390/pharmaceutics13050738/s1, Figures S1 and S2: Transmission electron microscopy of the 50 ppm AuNP-treated pericyte group.
