# Supplementary material for: Gold Nanoparticles Affect Pericyte Biology and Capillary Tube Formation
Source: Pharmaceutics. 2021 May 17;13(5):738. doi: 10.3390/pharmaceutics13050738 (PMC8156556; doi:10.3390/pharmaceutics13050738)
Supplement: Supplementary file 1 [file pharmaceutics-13-00738-s001.zip › pharmaceutics-1213886-supplementary.pdf]

pharmaceutics

Supplementary Information: Gold Nanoparticles Affect Pericyte Biology and Capillary Tube Formation

Sasikarn Looprasertkul, Amornpun Sereemaspun, Nakarin Kitkumthorn, Kanidta Sooklert, Tewarit Sarachana and Depicha Jindatip

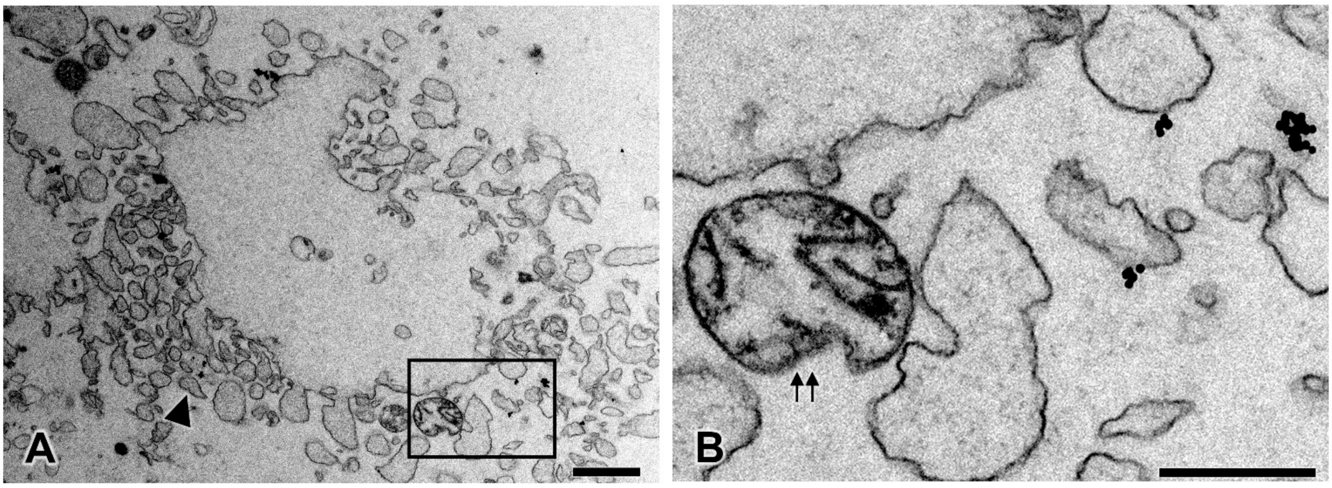

Figure S1. Transmission electron microscopy of the 50 ppm AuNP-treated pericyte group. A magnified view of the boxed area in **A** is shown in **B**. Some of the pericytes presented damaged plasma membranes, plasmalemmal blebs (*arrowhead*), swollen or damaged mitochondria (*double black arrows*) and free AuNPs (*electron-dense dots*) (**B**). Bars: 5000 nm (**A**), 1000 nm (**B**).

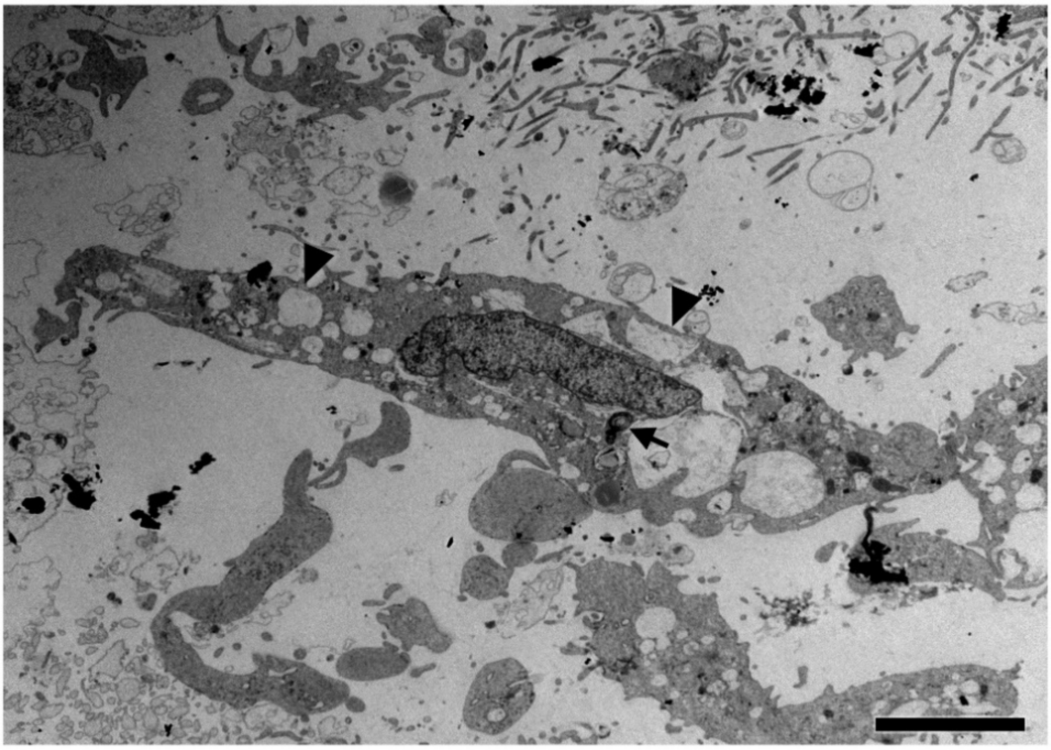

Figure S2. Transmission electron microscopy of the 50 AuNP ppm-treated pericyte group. Some of the pericytes presented continuous plasma membranes, numerous autophagic vesicles (*arrowhead*), and multilamellar whorl bodies (*black arrow*). Bar: 5000 nm.
